# Food supply and provisioning behavior of parents: Are small hoopoe nestlings condemned to die?

**DOI:** 10.1093/beheco/arad067

**Published:** 2023-09-12

**Authors:** Paula Ferrer-Pereira, Ester Martínez-Renau, Manuel Martín-Vivaldi, Juan José Soler

**Affiliations:** Calle Velázquez 74, Paracuellos de Jarama, 28860 – Madrid, Spain; Departamento de Ecología Funcional y Evolutiva, Estación Experimental de Zonas Áridas (EEZA-CSIC), Ctra. Sacramento S/N, La Cañada de San Urbano, 04120-Almería, Spain; Unidad Asociada Coevolución: Cucos, Hospedadores y Bacterias Simbiontes, Universidad de Granada, Avda. Funetenueva S/N, 18171-Granada, Spain; Departamento de Zoología, Facultad de Ciencias, Universidad de Granada, Avda. Fuentenueva S/N,18171-Granada, Spain; Departamento de Ecología Funcional y Evolutiva, Estación Experimental de Zonas Áridas (EEZA-CSIC), Ctra. Sacramento S/N, La Cañada de San Urbano, 04120-Almería, Spain; Unidad Asociada Coevolución: Cucos, Hospedadores y Bacterias Simbiontes, Universidad de Granada, Avda. Funetenueva S/N, 18171-Granada, Spain

**Keywords:** food allocation, food availability, nestling size hierarchy, parent–offspring communication, social context, *Upupa epops*

## Abstract

Parents might use signals of need or of quality to decide food provisioning among their offspring, while the use of one or another signal might depend on food availability. Begging success of nestlings of different quality (i.e., body size) would also depend on food availability, and we here explore the effect of experimental food supply in begging success of nestlings and in provisioning of female hoopoes (*Upupa epops*), a species with extreme hatching asynchrony and nestlings size hierarchy. We video-recorded food allocation of females, begging success of nestlings of different size, and the social context (i.e., the size category of the other nestlings that were begging for food) during periods when experimental food supply was or was not available in the same nests. We found that when experimental food supplementation was present, begging success of the intermediate, but not that of large or small-sized nestlings, increased. The experiment, however, did not affect the feeding preferences of females toward nestlings of different size. Moreover, when small nestlings were the only ones that were begging for food, their begging success decreased in the experimental period, and females used supplemented prey to feed themselves. Those results, on one hand, confirm the importance of food availability for the begging success of nestlings of particular sizes and, on the other, indicate that females prefer to use extra food for their own rather than for the smallest nestlings. We discuss possible mechanisms explaining the detected experimental effects and the adaptive and nonadaptive explanations of mothers ignoring the small nestlings.

## Teaser

Theoretically, parents should feed offspring that are begging for food; at least in situations of abundant resources. In accordance, during events of experimental food supply, medium-sized nestlings where feed more often. However, the begging behavior of small hoopoe nestlings were ignored by mothers even when only them were begging for food. In these situations, females used available prey to feed themselves. Possible functions of the existence of these condemned to die nestlings are discussed.

## INTRODUCTION

Reproduction is the most energetically demanding activity of animals, and reproductive success often depends on parents’ ability to nourish their offspring. In species where the parents take care of several offspring in a restricted space (i.e., nursery species [Mock and Parker 1997]), parents have to determine, not only the number of eggs to lay (Pincheira-Donoso and Hunt 2017), but also which offspring to invest in, and when and how much to invest in them (Clutton-Brock 1991; Caro, Griffin, et al. 2016; Li et al. 2019; Sechaud et al. 2022). In birds, when food availability is fully predictable, they use to lay clutches that produce broods that optimize the number of nestlings that the adults are capable of feeding (Lack 1947; Ricklefs 1980; Lima 1987; Vander Werf 1992). In contrast, when the availability of food is unpredictable, birds typically lay optimistic clutches that produce optimistic broods that, in case of limited availability of resources, parents can reduce or adjust to environmental conditions (Lack 1947, 1954, 1968). Brood reduction, however, does not occur randomly among siblings and the existence of asynchronous broods, in terms of, for instance, body size or competition skills, would facilitate brood size adjustment to environmental conditions (Lack 1947; Mock and Parker 1997; Barrionuevo and Frere 2017; Hildebrandt and Schaub 2018). The most common way for parents of producing a brood hierarchy is to start incubation before clutch completion, which results in asynchronous hatching and broods that include nestlings of different ages and sizes. Although hatching asynchrony might have adaptive and nonadaptive explanations, nestlings of small size are typically the object of brood reduction (Magrath 1990; Stoleson and Beissinger 1995; Soler, Ruiz-Raya, et al. 2022).

On the other side of the parent–offspring communication and conflict, by a variety of mechanisms, offspring typically try to influence parental behavior in their own benefit (Mock and Parker 1997). When begging for food, nestlings display specific vocal calls and elevate their open and flamboyant-colored mouths, showing to adults their level of need and their phenotypic and genetic quality (Kilner and Johnstone 1997; Caro, West, et al. 2016; Martínez-Renau et al. 2021). In general, the smaller nestlings beg for food more intensely than larger ones (Bengtsson and Rydén 1981; Price et al. 1996; Mock et al. 2011; Brode et al. 2021), but the responses of adults to the intense begging display of small nestlings show a continuous interspecific variation in the avian phylogeny (Caro, Griffin, et al. 2016). There are species that, as tree swallows (*Tachycineta bicolor*), preferentially feed the smaller begging nestlings (Leonard and Horn 1996, 2001), while some others, as hoopoes (*Upupa epops*), ignore the begging of small nestlings while eliciting bigger siblings to beg (Martín-Vivaldi et al. 1999). The substantial interspecific variation in parental responses to begging displays of nestlings is hardly explained by different species following the paradigms of either “signal of need” or “signal of quality” explaining parent-offspring communication and conflict. The “signal of need” models predict that parents should preferentially feed the offspring showing the highest level of need (Godfray 1991, 1995; Charlesworth et al. 2000; Mock et al. 2011). However, it does not apply to species where parents leave the neediest smallest offspring to starve. The “signal of quality” models would better explain that behavior, because they predict that, independently of the signal of need, parents would always prefer to feed offspring of higher probability of survival (i.e., bigger offspring) (Grafen 1990; Royle et al. 2002; Mock et al. 2011). Juveniles of most species, however, display characters reflecting phenotypic quality (i.e., size or body mass) and need (i.e., begging display), and their success attracting the attention of provisioning parents might depend on the interaction between their competitive abilities and need (Parker et al. 2002).

The relative importance of signals of need and of quality determining parental feeding preferences should also depend on environmental conditions and, thus, vary among species, among populations and even among nests within populations. In accordance, the interspecific variation in parental responses to the begging display of small nestling birds (i.e., signal of need) is related to the relative importance of signal of need and of quality in the begging behavior displayed by nestlings of different species (Caro, Griffin, et al. 2016). In an interspecific comparative study, Caro, Griffin, et al. (2016) showed that in bird species that typically breed in predictable and good environments, chicks in worse condition beg for food more intensely and parents preferentially feed them. Instead, in species that breed in unpredictable and poor environments and lay optimistic clutches, parents pay less attention to signals of need (i.e., begging) and, in the case of food limitation, they preferentially feed nestlings with higher reproductive value (e.g., those of larger body size) (Mock and Forbes 1995). Then, food availability or predictability has been claimed as one of the key factors explaining the extreme interspecific variation in begging behavior of nestling birds (i.e., nestling traits showing their quality or level of need honestly), and how parents respond to nestling signaling (Parker et al. 1989, 2002; Davis et al. 1999; Mock et al. 2011; Grodzinski and Johnstone 2012).

At the intraspecific level, food availability could also explain feeding behavior of parents in relation to begging display of nestlings. For instance, in species that usually experience brood reduction and in which parental feeding decisions rely mainly on signals of nestling quality, adults should change their strategy in a context of food abundance and attend to the signals of need of small nestlings (Caro, Griffin, et al. 2016); a prediction that, as far as we know, has never been tested experimentally. Even within the same nest, temporal variation in food availability could influence begging success of nestlings of different quality and feeding strategy of parents. During periods of food abundance, parents may differentially attend signals of need of intermediate or low-quality nestlings simply because those with the highest probability of survival would become rapidly satiated. In addition, during these periods of food abundance, even when only nestlings of the lowest quality beg for food, parents may decide whether to use the available food to feed small nestlings or use it for themselves. Here, we present the results of an experiment designed to test the influence of food availability on feeding decisions of parents and begging success of nestlings in a bird species: the hoopoe. We made extra prey temporally available at the end of the hatching period and monitored the provisioning behavior and self-feeding of the female in response to her offspring’s need (begging behavior) and quality (offspring size).

The hoopoe is a species with an extreme hatching asynchrony and nestling size hierarchy, in which females stay at the nests during incubation and hatching, and feed nestlings with prey delivered to the nest by the male (Martín-Vivaldi et al. 1999; Díaz-Lora et al. 2020; Diaz-Lora et al. 2021). In addition, it is worth to mention that females prefer to feed larger offspring (Martín-Vivaldi et al. 1999), that more than one nestling die soon after hatching in more than 70% of the nests (Ryser et al. 2016; Soler, Martín-Vivaldi, et al. 2022), and that brood reduction more often occurs in poor environments (Hildebrandt and Schaub 2018). Thus, following the hypothesis that food availability should affect feeding behavior of females and begging success of nestlings, we predicted that experimental food supply would increase begging success of small nestlings and feeding preference of females for them. We explored these predictions by comparing nests under different experimental treatments (i.e., among-nests comparisons), and by comparing periods when supplemental food was or was not available within each experimental hoopoe nest.

As it occurs for some other bird species (Ruiz-Castellano et al. 2016; Soler et al. 2017; Brode et al. 2021), food provisioning by female hoopoes will largely depend on social context (i.e., characteristics of the group of nestlings that are begging for food at a particular feeding event). Since we know that hoopoes prefer to feed larger offspring, the expected positive effects of extra food on feeding rates of small nestlings should mainly occur when larger nestlings are satiated and do not beg for food (Parker et al. 2002). Moreover, because female hoopoes also use prey carried by males to the nest to feed themselves, we explored the effect of food availability (i.e., experimental supplemented prey) on females’ decision of using the extra food for self-maintenance. In this case, we also analyzed the influence of social context (nestlings of different sizes that are begging for food) on females’ use of the food item for themselves or to feed begging nestlings; an influence that, as far as we know, has never been explored. We expected that, in situations of experimental food supply, mothers feed themselves primarily when all nestlings in the nests are satiated. In addition, when larger nestlings are not begging for food, females should use the extra prey to feed smaller begging nestlings, rather than to feed themselves.

## MATERIALS AND METHODS

### Study species

The hoopoe is a non-passerine migratory bird. Hoopoes are mainly insectivorous, live in small-scaled mosaic habitats of bare ground, and use areas without tall vegetation for feeding (Schaub et al. 2010; Tagmann-Ioset et al. 2012). They are cavity nesters that can equally use natural or artificial (nest boxes) cavities (Hoffmann et al. 2015). In our study area, the breeding season extends from February to July during which hoopoes can raise up to two broods, occasionally three, typically of six to eight eggs each (Martín-Vivaldi et al. 1999; Hoffmann et al. 2015; Plard et al. 2018). Females usually start to incubate with the first or the second laid egg (Cramp 1985; Martín-Vivaldi et al. 1999) and, because they lay one egg per day, this incubation pattern leads to relatively high levels of hatching asynchrony and nestling-size hierarchy, with the first hatched nestling being 6–7 days older than the last hatched one.

During the incubation and hatching periods (i.e., 7–8 days after the first egg hatched), the females rarely leave the nests and are mainly fed by the males (Díaz-Lora et al. 2020, 2021), who infrequently enter into the nests (Cramp, 1985). Thus, during the first 10 days after hatching the first eggs, the period when most brood reduction occurs (Hildebrandt and Schaub 2018), females have to decide, not only which nestlings to feed, but when to feed themselves with preys delivered to the nests by males (Díaz-Lora et al. 2020, 2021; Arco et al. 2022). Females use a single prey per feeding event.

### Study area and field procedures

The study population was located in southeast Spain, on the Guadix high altitude plateau (37°18ʹN, 38°11ʹW). There, the hoopoes breed in nest boxes (internal height × width × depth = 35 × 18 × 21 cm, bottom-to-hole height = 24 cm, and the entrance diameter = 5.5 cm) made of cork that are attached to tree trunks and walls, or hidden in piled stones. The fieldwork was carried out during the 2019 breeding season. Briefly, from early March to late June, nest boxes were visited every week until eggs were detected inside. Then, by assuming that hoopoes lay an egg per day, start the incubation with the second egg, and that incubation period lasts 17 days (Martín-Vivaldi et al. 1999), we estimated the expected hatching date and visited the nest 2–3 days later. Once the antepenultimate egg hatched, we commenced the experimental protocol, including the installation of the video recording equipment (see Electronic Supplementary Material 1 for details) and the food supplementation experiment (see below). Except for a few periods with technical problems with the video recording equipment, hoopoe nests were continuously recorded from sunrise to sunset.

**Figure 1:**
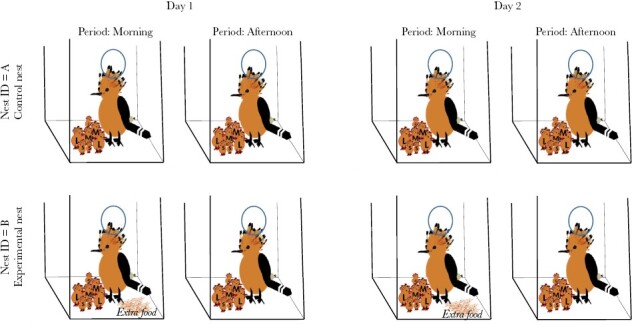
Diagram showing the experimental protocol. Control (no food added) and experimental nests (food added once per day) were recorded during 2 days for 2 day periods (morning and afternoon) and we analyzed the feeding behavior of females and the begging success of large (L) Medium (M) and small (S) nestlings.

**Figure 2:**
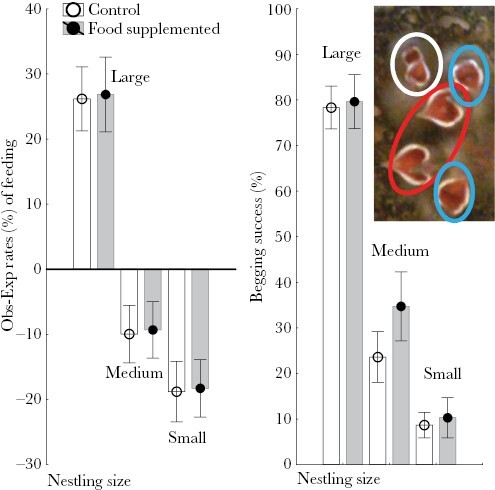
Comparisons of female preferences (observed minus expected rates (%)) and begging success of nestlings of different size category in experimental nests during recording events with and without extra-food added. Values are mean ± 95% CI. We also show a photogram where open mouths of small-, medium-, and large-sized nestlings can be seen within ellipses of white, blue, and red colors, respectively.

Hatching date was established as the day when the first hoopoe egg hatched, hatching span as the number of days that elapsed from hatching dates of the first to the last egg, and brood size as the maximum number of nestlings. At the beginning of the video recording periods, we weighed all nestlings in each nest with a digital balance (Ascher, accuracy 0.01 g), which allowed us to confirm nestling size categories (i.e., small, medium, and large) established from video recordings. Briefly, we first identified the largest-sized nestlings, that can be easily differentiated from smaller nestlings and, later, we differentiated those of smallest size that were certainly distinguished from larger siblings. The rest of nestlings were considered of intermediate size. Because the behavior of nestlings and females were analyzed for two periods per day, and during two consecutive days (see Figure 1), the same person (P.F.-P.) performed classification of nestlings within the three size categories (see Figure 2) for each visualization.

At the time of installing the video recording equipment, we randomly assigned experimental treatment to each nest. Experimental nests (*N* = 23) received approximately 20–25 (a filled Falcon tube of 15 mL) dead crickets (Genus *Gryllus or Acheta*) of different size (i.e., it included crickets of the three considered size categories (see below) (small: 5.41%, medium: 79.28% and large: 15.32%; *N* = 666) that were used by females to feed different sized offspring and themselves. Crickets are a common (Fournier and Arlettaz 2001; Hoffmann et al. 2015; Guillod et al. 2016; Ryser et al. 2016) and preferred (Arco et al. 2022) prey of hoopoes. Commercial fresh crickets were stored in the freezer at −20 °C for a maximum of 15 days and defrosted for at least 3 h at room temperature before introducing them into the nests. Females rapidly detected and used them to feed their offspring and themselves. Experimental crickets lasted about 1 or 2 h. Experimental and control nests (*N* = 24) were daily visited to renew the batteries and SD memory cards, and to refresh the food supplemented treatment by emptying the Falcon tube containing crickets through the nest-box entrance.

The food supplementation experiment started 1 or 2 days before the last hoopoe egg hatched and extended until the death of one or two nestlings occurred, or the second or third day after the end of hatching. Then, the experiment lasted a maximum of 5 days and, because supplemented food was usually consumed within the first 2 h, hoopoes in experimental nests experienced several daily periods when experimental food was or was not available in the nest (see Figure 1). Thus, in experimental nests, we were able to explore differences in the provisioning behavior of the same female and the begging behavior of her nestlings during periods when food supply was or was not available. We analyzed around 20 consecutive feeding events (mean = 20.21, Min = 12, Max = 29) for each of the considered recording periods (i.e., the morning and the afternoon [5 or more hours later] of two consecutive days, see Figure 1).

### Analyzing and estimating begging behavior of nestlings and food allocation of female hoopoes

For each video-recorded nest, we (P.F.-P.) characterized food allocation patterns of the female and the begging behavior of nestlings of different sizes in the brood hierarchy. Because nestlings of all different sizes begged for food in most of the recorded feeding events (see Results), feeding preferences of females toward nestlings of each size category was estimated as the deviations (i.e., observed [OBS] minus expected [EXP] values) of feeding rates from those expected under the assumption of evenly distribution of food among siblings of different size. For instance, if two out of five siblings in a nest are of medium size, an evenly distribution of food would predict that they will receive 40% of the feedings (EXP) and, if they were fed at a rate of 15% (OBS), feeding preference of females would be estimated as -25%.

Begging rates were estimated as the percentage of feeding events in which nestlings of each size category beg for food. Moreover, by considering feeding events where nestlings of a particular size category begged for food, begging success was estimated as the rate (in percentage) in which begging nestlings of each size category were fed by their mother. We also estimated feeding speed as the number of observed feeds divided by the time in hours of each analyzed recording period. Finally, also from video recordings, we estimated the size of each prey item as being shorter than ¼ (size = 1), between ¼ and ½ (size = 2) or larger than ½ (size = 3) the length of the mother’s beak. We did so for experimental (crickets) and natural food items.

### Statistical procedures

The effects of day period (morning vs. afternoon), and recording day (first vs. second) (see Figure 1) on feeding preferences of females toward nestlings of different sizes, or on begging success of nestlings of different size category, were explored in control nests with information of nestlings of the three categories (*N* = 22) by mean of repeated-measures ANOVAs. Values for nestlings of each of the three considered size categories were used as the repeated measure, and day, day period and nest identity as the between factors. The female’s preference (i.e., repeated measures) did not depend on the day (interaction with nestlings’ size category; *F* = 1.99, df = 2, 98 *P* = 0.14) or on the day period (interaction with nestlings’ size category; *F* = 2.48, df = 2, 98, *P* = 0.089) of the recoding event. Similarly, begging success of nestlings of different sizes did not depend on the day (interaction with nestlings’ size category; *F* = 0.270, df = 2, 98, *P* = 0.764) or the day period (interaction with nestlings’ size category; *F* = 2.15, df = 2, 98, *P* = 0.122) of the recording events. Finally, both female preferences (*F* = 4.32, df = 2, 98, *P* < 0.0001) and begging success (2.90, df = 2, 98, *P* < 0.0001) of nestlings of different size varied among recorded hoopoe nests. Thus, in subsequent analyses, for nestlings of different size categories, we used average values of the recording periods in control nests, while for experimental nests, we separately averaged values of periods with and without extra-food in the nest.

Among-nests variation in feeding preferences of females or begging success of nestlings of different size categories due to experimental food supply were also explored in RMAs. In this case, for control nests, we used a single average value per nest that always corresponded to periods with no extra-food (*N* = 22). For experimental nests, we used two average values per nest that respectively corresponded to periods with and without extra food. Because we only used nests that contained nestlings of the three different size categories, and in experimental nests some nestlings died (small) and some others changed their size-category position from one to the next analyzed period (e.g., from medium to small), sample size of experimental nests differed for periods with (*N* = 22) and without (*N* = 19) food supplementation. Thus, we separately compared control nests with experimental nests in situations where extra-food was or was not available. These models included feeding preferences of females or begging success of nestlings of the three different size categories as the repeated measures, experimental treatment as the discrete between-factor, and laying date, brood size and speed of feeding as continuous between-factors. Continuous between-factors that did not reach statistical significance were one by one removed from the statistical models.

The within-nest variation in feeding preferences of females or begging success of nestlings of different size categories due to the presence or absence of experimental food supply (i.e., only experimental nests can be considered) were also analyzed by RMAs. In this case, we compared values of food allocation of females and begging success of nestlings estimated for each nestling size category (first repeated measures), and for periods when extra food was or was not available for feeding (second repeated measures). Significant interactions between these two repeated measures (within factors) will inform on differential effects of experimental food on nestlings of different size. Similarly, the effect of extra-food on rate of prey used by females to feed themselves was also estimated in RMAs with extra-food as the only within factor. The statistical program used to run those RMAs was STATISTICA 12 (Dell-Inc., 2015).

We also explored the possible effects of social context in interaction with food availability (i.e., experimental food addition) on whether nestlings of different size category that were begging for food were or were not fed by females. To do so, we analyzed the 2944 feeding events that were recorded in control and experimental nests with nestlings of the three size categories (*N* = 43). For each feeding event, we noted whether it occurred during recording events with extra-food, the size of the nestling that received the food item, and the social context (i.e., size categories of nestlings that were begging for food). The social context therefore includes eight possibilities: (1) large, medium, and small; (2) large and medium; (3) large and small; (4) only large; (5) medium and small; (6) only medium; (7) only small; or (8) none of the nestlings, were begging for food. Thus, since we are interested on exploring the effect of social context and experimental food supplementation on begging success of smaller, medium and larger nestlings, we used separated Generalized Linear Mixed Models (GLMM) with a binomial distribution (begging success [yes or no]) to test the effects of such fixed factors on the begging success of nestlings of different size. The models also included nest identity as random factor (i.e., intercept random effect), and social context (whether nestlings of a target size category beg for food alone or together with nestlings of other size categories) and food supplementation experiment (recording event with or without supplementary food available in the nest) as the fixed effects. Moreover, because the size of prey items could also determine food allocation decisions of females, prey size was also included as a continuous predictor in the GLMMs. The interaction between social context and experimental food supply was estimated in separate models that also included main effects, and will inform on whether the effect of social environment depends on food availability. Similar GLMMs were used to estimate the effects of social environment and food availability on females’ decisions of using prey to feed themselves. We performed these analyses in R-environment (version 3.6.1) with the library GLMMTMB (Brooks et al. 2017).

## RESULTS

During the experimental period, nestling deaths occurred in 33 out of the 47 nests (70.21%), and the intensity of brood reduction in video recorded nests typically varied between 1 and 2 nestlings (95% confidence interval [CI] = 0.86–1.48). Neither prevalence nor intensity of brood reduction differed between control and experimental nests (Supplementary Material 2). Thus, our experiment did not affect the survival prospect of nestlings, but only temporal availability of extra-food for feeding females. As expected, feeding rates of nestlings drastically increased during periods of food supply (log-transformed feeding events to nestlings per hour: control period (95% CI): 2.04–2.38, food supply period (95% CI): 2.92–3.68; RMA, *F* = 32.80, df = 1, 22, *P* < 0.0001), which confirms that our experimental approach affected food availability for feeding. Otherwise, neither clutch size, brood size at hatching, hatching failure, number of large, medium and small nestlings, nor nestling hierarchy (difference in body mass between the first and the last hatched nestling at the end of hatching) differed significantly between experimental and control nests (*P* > 0.40, See Supplementary Material 2).

### Food allocation of females and begging success of nestlings of different size categories in experimental and control nests

#### Between-nest comparisons

We analyzed 336 h and 34 min of video, and recorded 3617 feeding events in 47 hoopoe nests. In those that included all three nestling size-categories (small, medium, and large nestlings) (43 nests, 21 experimental and 22 control; total feeds = 2944), most feeds were obtained by nestlings of larger size (61.11%) followed by medium nestlings (21.10%). In those nests, the smallest nestlings were only sporadically fed (6.49%), while females used to feed themselves (11.31%) with prey items. Moreover, in most of those recorded feeding events nestlings of different size begged for food (small = 73.00%, medium = 71.74%, and large = 78.67%). The female’s preference and begging success were associated with nestling size, while the effects of experimental treatment were far from statistical significance (Table 1). That was the case regardless of considering or not periods when food supply was available in experimental nests (Table 1), or of controlling for the effects of hatching date, brood size, and feeding speed (Results not shown).

**Table 1:** 95% Confidence intervals (± 95% CI) of least square means of female preferences (observed minus expected values from an evenly distribution of food among nestlings of different size) and begging success of nestlings of different size categories in control and experimental nests. Comparisons between control and experimental nests were separately done for periods when experimental food was or was not available in experimental nests. Results from repeated-measures ANOVAs that included nesting size category as repeated-measures factor and experimental treatment as between factor are shown.

	Control nests (*N* = 22)			Experimental nests (*N* = 19, *N* = 22)			Effects			
	Large	Medium	Small	Large	Medium	Small	Nestling size		Nestling size × Exp treatment	
	± 95% CI	± 95% CI	± 95% CI	± 95% CI	± 95% CI	± 95% CI	*F*(2, 78)	*P*	*F*(2, 78)	*P*
No extra food available in Experimental nests										
Female preference	28.5–19.5	−4.2 to −11.8	−16.8 to −24.9	31.4–21.7	−6.30 to 14.4	−13.7 to −22.3	175.5	<0.0001	0.78	0.508
Begging success	82.3–72.6	36.2–23.3	10.9–4.4	83.2–72.8	31.16–17.3	12.7–5.6	366.6	<0.0001	1.05	0.355
Extra food available in experimental nests							*F*(2, 84)	*P*	*F*(2, 84)	*P*
Female preference	28.7–19.2	−4.5 to −11.5	−16.7 to −23.5	30.2–20.6	−5.07 to 12.1	−15.3 to −22.5	181.9	<0.0001	0.11	0.893
Begging success	82.6–72.3	36.8–22.7	11.6–3.7	85.4–75.0	42.3–28.2	13.2–5.3	366.1	<0.0001	0.29	0.752

#### Within-nest comparisons

Females in experimental nests that harbored nestlings of the three size categories with information for periods with and without extra food (*N* = 19) allocated food preferentially to large nestlings, while almost ignoring the smallest ones (Figure 1), resulting in highly statistically significant influences of nestling size category (RMA, *F* = 118.00, df = 2, 36, *P* < 0.0001). That bias in food allocation did not change in periods of experimental food supply (RMA, interaction between supplemental food availability and nestling size categories, *F* = 0.15, df = 2, 36, *P* = 0.864). When focusing on begging success of nestlings, it increased during periods of supplemental food (RMA, *F* = 5.44, df = 1, 18, *P* = 0.031). This tendency was mainly due to the change in begging success experienced by nestlings of medium size (Figure 1, RMA, interaction between supplemental food availability and nestlings’ size category, *F* = 5.41, df = 2, 36, *P* = 0.008; Figure 1).

**Figure 3:**
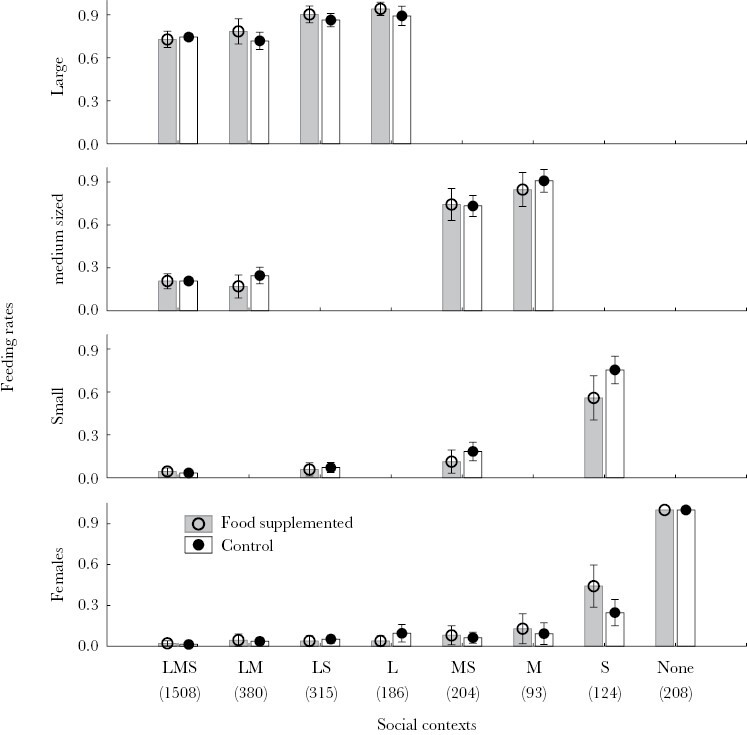
Mean ± 95% CI of provisioning rates of females to themselves and to large- (L), medium-sized (M), and small-sized (S) begging nestlings depending on social context (i.e., kind of nestlings that beg for each particular prey item). For example, LMS refers to feeding events (i.e., food items), where all, large-, medium-, and small-sized nestlings, beg for food, while S refers to feeding events where only small nestlings beg). Numbers under each of the eight possible social contexts indicate the number of feeding events that occurred in each social context.

As predicted, success of nestlings of different size that beg for a particular food item depended on nest-social context (size category of nestlings that beg for a particular food item) (Table 2). Whether or not nestlings of larger size were also begging for food was the main determinant (Figure 3). Interestingly, although the experimental food supplementation in interaction with social context did not predict begging success of nestlings of different sizes (Table 2), begging success of small nestlings decreased when only nestlings of this size begged (GLMM, χ^2^ = 4.04, df = 1, *P* = 0.044, Figure 3). That was the case after controlling for the nonsignificant effects of prey size (χ^2^ = 0.88, df = 1, *P* = 0.347) and the random variance due to nest identity.

**Table 2:** Results from GLMMs exploring the effects of the food supplementation experiment (extra-food), social context (size category of nestlings that were begging for food at a particular feeding event) and prey size, on the probability of obtaining food when begging for a particular food item. The models included begging success of large, medium and small nestlings as the binomial response variables, extra food, social context and prey size as independent fixed factors and nest identity as the random factor. The interactions between extra food and social context were explored in separate models that also included main effects. We also show results from similar models explaining female decision of feeding themselves when some nestlings were begging for food (females feeding themselves). Effects lower than 0.1 were highlighted in bold.

	Extra-food (E)			Social context (S)			Prey size			Interactions (E × S)		
	χ^2^	df	*P*	χ^2^	df	*P*	χ^2^	df	*P*	χ^2^	df	*P*
Begging success of large nestlings	0.193	1	0.660	**50.87**	**3**	**<0.00001**	0.232	1	0.630	5.317	3	0.150
Begging success of medium size nestlings	0.679	1	0.410	**260.82**	**3**	**<0.00001**	0.539	1	0.463	5.082	3	0.165
Begging success of small nestlings	2.507	1	0.113	**256.50**	**3**	**<0.00001**	0.224	1	0.569	5.278	3	0.153
Females feeding themselves	2.390	1	0.158	**108.07**	**6**	**<0.00001**	1.901	1	0.168	5.939	6	0.430

### Females using resources to feed themselves

On average, females consumed 5.27% (95% CI = 2.93–7.60) of the available food items during recording events (20 preys on average) with no extra food added (*N* = 47 nests). In recording events with extra-food added (*N* = 23 nests), it increased to 30.02% (95% CI = 18.72–41.32), which resulted in a highly significant effect of food supply in experimental nests (RMA, *F* = 24.32, df = 1, 22, *P* = 0.00006). Moreover, prey consumption by females also depended on nest social context (Table 2). They mainly consumed prey when none or only small offspring were begging for food (Figure 3); a pattern that was intensified during periods of food supplementation. During such experimental periods, prey consumption by females obviously increased exactly at the same rate that begging success of small nestlings decreased (GLMM, χ^2^ = 4.04, df = 1, *P* = 0.044, Figure 3). That was the case after controlling for the nonsignificant effects of prey size (χ^2^ = 0.88, df = 1, *P* = 0.347) and the random variance due to nest identity.

## DISCUSSION

Here, we experimentally tested the effects of food availability at the end of the hatching period on food allocation patterns of female hoopoes and begging success of nestlings of different sizes. Contrary to the expectations, the female’s preference toward small nestlings, or the begging success of small nestlings, did not differ between experimental and control nests, or between periods when food supply was or was not available in experimental nests. Rather, begging success of medium-sized nestlings, but not the female’s preferences toward them, increased in periods of food supply in experimental nests. Moreover, independently of the size categories of nestlings that begged for food in particular feeding events (i.e., social context), feeding rates of large- and medium-sized nestlings did not depend on experimental food availability, and, contrary to the expectations, feeding rates of small-sized nestlings decreased in experimental periods when they were the only begging offspring in the nests. In these conditions, females primarily used food to feed themselves instead of feeding starving small nestlings. These results suppose weak-partial support to the predicted effects of food availability on food allocation of adults among offspring, but they support the hypothetical effects on begging success of nestlings of a particular size. Below, we discuss alternative explanations and implications of our results for understanding the effect of food availability on parent–offspring interactions in species such as hoopoes with extreme asynchronous hatching.

With high food availability, females should pay more attention to signals of need and, thus, smaller nestlings should be fed more often, and their begging efforts more frequently rewarded in those situations (Caro, Griffin, et al. 2016). Another mechanism predicting an increase in feeding rates of smaller nestlings during periods of food abundance is that larger nestlings became rapidly satiated, which would shortly convert medium-sized nestlings into the preferred nestlings for feeding females. Several arguments suggest that the later explanation adjusts better to our results. Our experimental food supplementation temporary increased feeding rate of nestlings in general, but it did not affect feeding preferences of females. Thus, the detected temporary increase of begging success of medium nestlings may be the consequence of satiation of larger nestlings due to the increased feeding rates during periods of food abundance. Moreover, although we cannot completely rule out the possibility that the experiment was not long enough to induce detectable changes in the feeding behavior of females, feeding preferences and begging success of nestlings of different sizes did not differ among experimental and control nests. This result suggests that the detected increase in begging success of medium nestlings is a short-term effect of temporary abundant food supply. Indeed, there was a highly significant effect of social context on food allocation of female hoopoes. Nestlings of medium size had greater success when they competed for food with only nestlings of smaller size. Therefore, while experimental results confirm a role of food availability in determining the begging success of nestlings of different size category, these effects are unlikely explained by food abundance inducing a change in food allocation rules of hoopoe females, but by provoking rapid satiation of large nestlings.

Preferential feeding of females for larger nestlings might seem contradictory to previous results published by Ryser et al. (2016), where they demonstrated that females are more selective than male hoopoes when it comes to food allocation to the smallest chick. However, those authors studied food allocation by adult hoopoes after most of the brood reduction had occurred (i.e., when females leave the nests and together with males bring food to feed leftover nestlings) and, then, it is possible that food allocation behavior of females changes at that nestling stage (see also Soler, Martín-Vivaldi, et al. 2022a).

The hypothesis of food availability determining food allocation of adults, was mainly directed to understand why adults sometimes ignore signals of need of their offspring and concentrate on feeding nestlings of better survival prospect and, therefore, with higher reproductive value. Thus, expected changes caused by experimental food supply in food allocation patterns and begging success are typically focused on the smallest nestlings. However, contrary to the expected positive effects of food supply on small nestlings, we found that, in periods of food abundance, their feeding rates decreased when they were the only nestlings that begged for food. In these situations, females preferred to use extra-prey to feed themselves, which strongly suggest that smaller nestlings have almost nil probability of survival. Hoopoes produce asynchronous broods with a pronounced size hierarchy with nestlings of intermediate size that, together with smallest nestlings, might also starve due to females ignoring their signals of need (Martín-Vivaldi et al. 2014; Soler, Martín-Vivaldi, et al. 2022). Consequently, it might be possible that the tested hypothesis applies to hoopoe nestlings of intermediate size and, thus, the expected effects of food availability were only detected in that kind of nestlings. However, it is also possible that the provided experimental food supply was not abundant enough to satiate both large and medium nestlings, and that females prefer to use experimental food for themselves instead of using it for nestlings that will be ignored later, when food supply ends.

Whatever the explanation, ignoring small nestlings in periods of food abundance when they were the only nestlings that begged for food is an unexpected result that deserves discussion. Starvation of the smallest nestlings independently of food availability has been previously described in hoopoes (Martín-Vivaldi et al. 2014; Hildebrandt and Schaub 2018), and also occurs in groups such as hornbills (e.g., Carstens et al. 2019), raptors (e.g., Margalida et al. 2007), or penguins (e.g., Stein and Williams 2013), which systematically lay two or three eggs, but only raise one nestling. Thus, ignoring nestlings that beg for food may be spread in the avian phylogeny, particularly in species with extreme hatching asynchrony and extreme nestling size hierarchy.

Which is the functionality of such nestlings or of last laid eggs? The high rates of hatching failures in hoopoe nests, which in our study area are close to two eggs on average (Martín-Vivaldi et al. 1999; Soler et al. 2008, Soler, Martín-Vivaldi, et al. 2022) might suggest an insurance function of the last laid eggs (Forbes 1990, 1991; Hardy 1992). This is likely the case because the intensity of brood reduction, that we detected up to 2 days after the end of hatching, associated negatively with hatching failures (β(SE) = −0.365 (0.143), *F* = 6.50, df = 1, 44, *P* = 0.014), even after controlling for the effect of hatching date (β(SE) = 0.287 (0.143), *F* = 4.02, df = 1, 44, *P* = 0.051). In cases where no hatching failures occurred, the value of the last hatched nestlings is reduced and it might be better to lose the initial price of laying an extra egg than feed extra nestlings at the cost of older siblings (Forbes 2009); a scenario that might account for our unexpected results. Laying extra eggs might, however, have multiple functionalities (Mock and Parker 1986) and the presence of condemned-to-death nestlings in the brood, besides of being the result of reduced hatching failures, might increase fitness prospects of their older siblings. That would be the case if, for instance, they serve as food for older siblings (Alexander 1974), a possibility for which strong support in hoopoes has been published recently (Soler, Martín-Vivaldi, et al. 2022). Consumed small nestlings might then allow recovering part of the initial price of laying extra eggs that could have been laid as insurance. However, the rate of brood reduction is typically twice that of hatching failures (Soler, Martín-Vivaldi, et al. 2022) and, thus, it is likely that the fact that females used small nestlings to feed larger ones allows more optimistic clutch sizes, with some of the last hatched nestlings having a nutritive function. In this scenario, the lower feeding rates of small nestlings during periods of food supply could be interpreted as females trying to maintain small surplus nestlings alive during some days with the minimum and constant feeding rate per unit of time. By doing that, females could maintain “fresh food” in the nests and use small nestlings later, during periods of food scarcity.

Summing up, we found partial support to the expected effect of food availability determining begging success of nestlings of different sizes. We also found strong support to the role of social context determining begging success, but contrary to the hypothetical role of food availability influencing food allocation patterns, begging success of smallest nestlings decreased in periods of food supplementation. This last result leaves open the question of the adaptive significance of last hatched nestlings that are often condemned to death. Future research should focus on determining possible functions of these small nestlings of apparent surplus.

## Supplementary Material

arad067_suppl_Supplementary_Material

## Data Availability

Analyses reported in this article can be reproduced using the data provided by Ferrer-Pereira et al. (2023).

## Notes

We thank Manuel Soler Cruz for his comments on a preliminary version of the manuscript. We are very grateful to Scott Forbes and an anonymous Reviewer for their comments and suggestions that have increased the quality of the manuscript. Alberto Ruiz Moreno and Enrique Cortés Sánchez helped us with the recording equipment. Natalia Juárez García-Pelayo, Cristina Ruíz Castellano and Lola Barón helped us with fieldwork.
