# Supplementary material for: Food supply and provisioning behavior of parents: Are small hoopoe nestlings condemned to die?
Source: Behav Ecol. 2023 Sep 12;34(6):992–1001. doi: 10.1093/beheco/arad067 (PMC10636730; doi:10.1093/beheco/arad067)
Supplement: arad067_suppl_Supplementary_Material [file arad067_suppl_supplementary_material.docx]

**ELECTRONIC SUPPLEMENTARY MATERIAL**

**ESM 1: Video recording and equipment installation**

For video recording, we used mini video-cameras (JCHENG Mini Hidder surveillance camera) with the infrared filter removed by the technique services of the EEZA-CSIC. This allows recording under dim light and with an external source of infrared light (i.e. not detected by hoopoes nor human eyes). Cameras were tied to a rigid wire and placed at the top of the experimental nest boxes. An external screen (KKMoon 3,5” TFT LED, OWSOO-EU) plugged to the camera helped us to check that the camera works and that the scene includes the nest bottom where hoopoe nestlings were. The camera was also connected to an external recorder (mini_dvr, eBoTrade) with a 32MB SD card that allowed recording approximately 10 hours in high quality and more than 24 hours in low-medium quality. We also employed 12V external batteries (12AH) to maintain the camera and the recorder working for such long periods. During the second part of the breeding season, we used a power relay module with adjustable timing cycle (Walfront9wf1cd48go, ASIN B074VVKW4K) to turn off the gadgets. All the supplies were protected inside plastic bags (except the wires) and camouflaged with natural materials, either under the nest boxes or around the tree or wall. Finally, we used infrared light connected to another small battery that were camouflaged and sited on the top of the nest box. The mothers were often inside the nest box when the installation was going to be made. In those cases, the hoopoe was taken and put aside in a cotton bag while the installation procedure and then released into the nest again. Sometimes the mother left the box once she was freed but came back after a few minutes and began to behave normally again (brooding and feeding the chicks or cleaning the nest).

**EMS 2:** Average and 95% Confidence Intervals (CI) of clutch size, brood size at hatching, hatching failure, brood reduction (intensity and prevalence) that occurred in hoopoe nest up to two days after hatching of the last eggs. Information on number of large, medium and small nestling during recording events, and nestling hierarchy (difference in body mass between the first and the last hatched nestling at the end of hatching) of experimental and control nests is also included. Results from comparisons (one-way ANOVAs (F_1,45_) or Chi-square (Χ_1_) tests) between experimental and control nests are also shown

|  |  | Control nests  (N = 24) |  | Experimental nests  (N = 23) | F_1,45_  Χ_1_ | P |
| --- | --- | --- | --- | --- | --- | --- |
|  |  | Mean (95% CI) |  | Mean (95% CI) |  |  |
| Clutch size |  | 7.25 (6.80 – 7.70) |  | 7.39 (6.91 – 7.87) | 0.19 | 0.660 |
| Brood size at hatching |  | 6.21 (5.56 – 6.86) |  | 6.52 (5.94 – 7.10) | 0.55 | 0.461 |
| Rate of hatching failure of eggs |  | 1.04 (0.64 – 1.44) |  | 0.87 (0.43 – 1.31) | 0.36 | 0.552 |
| No. of large nestlings |  | 2.50 (2.24 – 2.77) |  | 2.66 (2.29 – 3.03) | 0.51 | 0.479 |
| No. of medium nestlings |  | 1.67 (1.31 – 2.04) |  | 1.80 (1.38 – 2.21) | 0.22 | 0.642 |
| No. of small nestlings |  | 1.36 (1.17 – 1.55) |  | 1.41 (1.08 – 1.75) | 0.09 | 0.766 |
| Brood reduction intensity  (2 days after hatching the last eggs) |  | 1.38 (0.88 – 1.87) |  | 0.96 (0.58 – 1.34) | 1.91 | 0.174 |
| Brood reduction prevalence  (2 days after hatching the last eggs) |  | 75.00 % |  | 65.22 % | 0.54 | 0.46 |
| Differences in body mass of the first and last hatched nestlings at the end of hatching |  | 31.74 (28.00 – 35.51) |  | 34.38 (29.15 – 39.62) | 0.72 | 0.40 |
| Hatching span |  | 5.71 (5.02 – 6.40) |  | 6.09 (5.45 – 6.72) | 0.70 | 0.41 |
